# Is physical therapy effective following extracorporeal shockwave lithotripsy and retrograde intrarenal surgery: a meta-analysis and systematic review

**DOI:** 10.1186/s12894-020-00664-9

**Published:** 2020-07-09

**Authors:** Linjie Peng, Junjun Wen, Wen Zhong, Guohua Zeng

**Affiliations:** 1 Urology, the First Affiliated Hospital of Guangzhou Medical University, Kangda Road 1, Haizhu District, Guangzhou, 510230 China; 2 Guangdong Key Laboratory of Urology, Kangda Road 1, Haizhu District, Guangzhou, 510230 China; 3 Guangzhou Institute of Urology, Kangda Road 1, Haizhu District, Guangzhou, 510230 China; 4 Guangzhou Medical University, Guangzhou, China

**Keywords:** Physical therapy, External physical vibration lithecbole, Percussion, Inversion, Lithotripsy, Stone free rate, Complications

## Abstract

**Background:**

Physical therapy, including percussion, inversion, vibration and combinations, was clinically performed to improve the stone free rate (SFR) following lithotripsy procedures. However, physical therapy is not widely accepted in clinical practice owing to lack of high level evidence support and a standard protocol. The present meta-analysis aimed to evaluate the efficacy and safety of physical therapy in improving SFR following extracorporeal shockwave lithotripsy (ESWL) and retrograde intrarenal surgery (RIRS).

**Methods:**

Systematic review of literature from PubMed, Scopus, Cochrane library and Embase was performed in March 2019. The efficacy and safety of physical therapy after ESWL and RIRS were assessed by meta-analysis of SFR and complication rate.

**Results:**

A total of 8 prospective studies with 1065 patients were enrolled. When compared to non-intervention, physical therapy provided a higher SFR (OR:3.38, 95% CI: 2.45–4.66, *p* < 0.0001) at all time points (week 1, week 2 and month 1), while there was no significant difference in complications such as hematuria, lumbago, dizziness and urinary tract infection (OR: 0.84; 95%CI: 0.62–1.13; *p* = 0.237). In subgroup analysis of different stone locations, lower calyx stone (OR: 3.51; 95%CI: 2.21–5.55; *p* < 0.0001), upper ureter and renal pelvic stones (OR:2.79; 95%CI:1.62–4.81; *p* = 0.0002) had a higher SFR after physical therapy, while there was no significant improvement in SFR in upper and middle calyx stones. In subgroup analysis of different techniques, EPVL (external physical vibration lithecbole, OR:3.47; 95%CI:2.24–5.37; *p* < 0.0001) and PDI (percussion, diuresis and inversion, OR:3.24; 95%CI:2.01–5.21; *p* < 0.0001) were both effective in improving SFR when compared to non-intervention.

**Conclusions:**

Physical therapy is effective in improving the SFR after ESWL and RIRS, especially for lower calyx stones, upper ureter and renal pelvic stones, while without significant side effects. External physical vibration lithecbole (EPVL) might provide a relative uniformed and repeatable protocol for clinical practice of physical therapy.

**Trial registration:**

PROSPERO 2019 CRD42019130228.

## Background

Urolithiasis is one of the most frequently noted diseases in urology. The incidence of urolithiasis varies from 1 to 13% in different area, and is still increasing [1, 2]. Percutaneous nephrolithotomy (PCNL) is the first line choice for calculus larger than 2 cm, while extracorporeal shockwave lithotripsy (ESWL) and retrograde intrarenal surgery (RIRS) are well established procedures for moderate size stones ranged from 1 cm to 2 cm [3–5]. The essential characteristics of PCNL, ESWL and RIRS destine different stone free rate (SFR) and complication rate. Accordingly, PCNL, ESWL and RIRS have their inherent position in the management of upper urinary tract stones [6–11].

When compared to PCNL, the SFR after ESWL and RIRS is getting increasingly concerned, since spontaneous passage of stone fragments following ESWL and RIRS is more required than PCNL. It has been reported that SFR ranges from 23.1 to 91.5% and 45.6 to 96.7% in ESWL and RIRS, respectively [12]. Residual stone fragments related complications are foreseeable, urinary tract infection, renal colic and steinstrasse are most common and might require additional intervention [13, 14]. Furthermore, with a recurrence rate of 50% within 5 years and 80–90% within 10 years, residual stone fragments are more prone to recurrent and thus bring great economic burden [15].

Medical expulsive therapy (MET), including diuretics, Chinese patent medicine and α receptor blockers (tamsulosin), have been used as auxiliary method to improve SFR following lithotripsy [16]. However, the medicine effect of tamsulosin in dilating ureter and facilitating stone fragments passage is controversial [17–19], as well as other medicines.

Theoretically, lower calyx stones (LCS) are more prone to stay in situ owing to the gravity and renal collecting system anatomy, especially when patients keep a vertical position [20]. Stone fragments rolling into ureteral pelvic junction or upper ureter due to body position change would increase the possibility of self-expulsion [21]. Thus, self-help position therapy has been performed to improve SFR after lithotripsy [22, 23]. Interestingly, physical activities like roller coaster and intercourse have been proved to promote renal stones expulsion, indicating a potential modality to get a higher SFR [21, 24–28]. Later, physical therapies including percussion and inversion were tried in clinical practice [29]. More recently, external physical vibration lithecbole (EPVL) was designed to facilitate stone fragments passage, which combined the vibration and inversion by a precise manipulation from a machine made in China [30].

Unfortunately, there are currently no conclusive evidences of physical therapy in facilitating stone fragments passage after lithotripsy, nor uniformed or widely accepted protocols of physical therapy. The present meta-analysis was aimed to evaluate the role of physical therapy in improving SFR following ESWL and RIRS, and provide a high level evidence for urologists to take physical therapy into serious consideration when to improve SFR following ESWL and RIRS.

## Methods

### Literature search and article selection

**Fig. 1:**
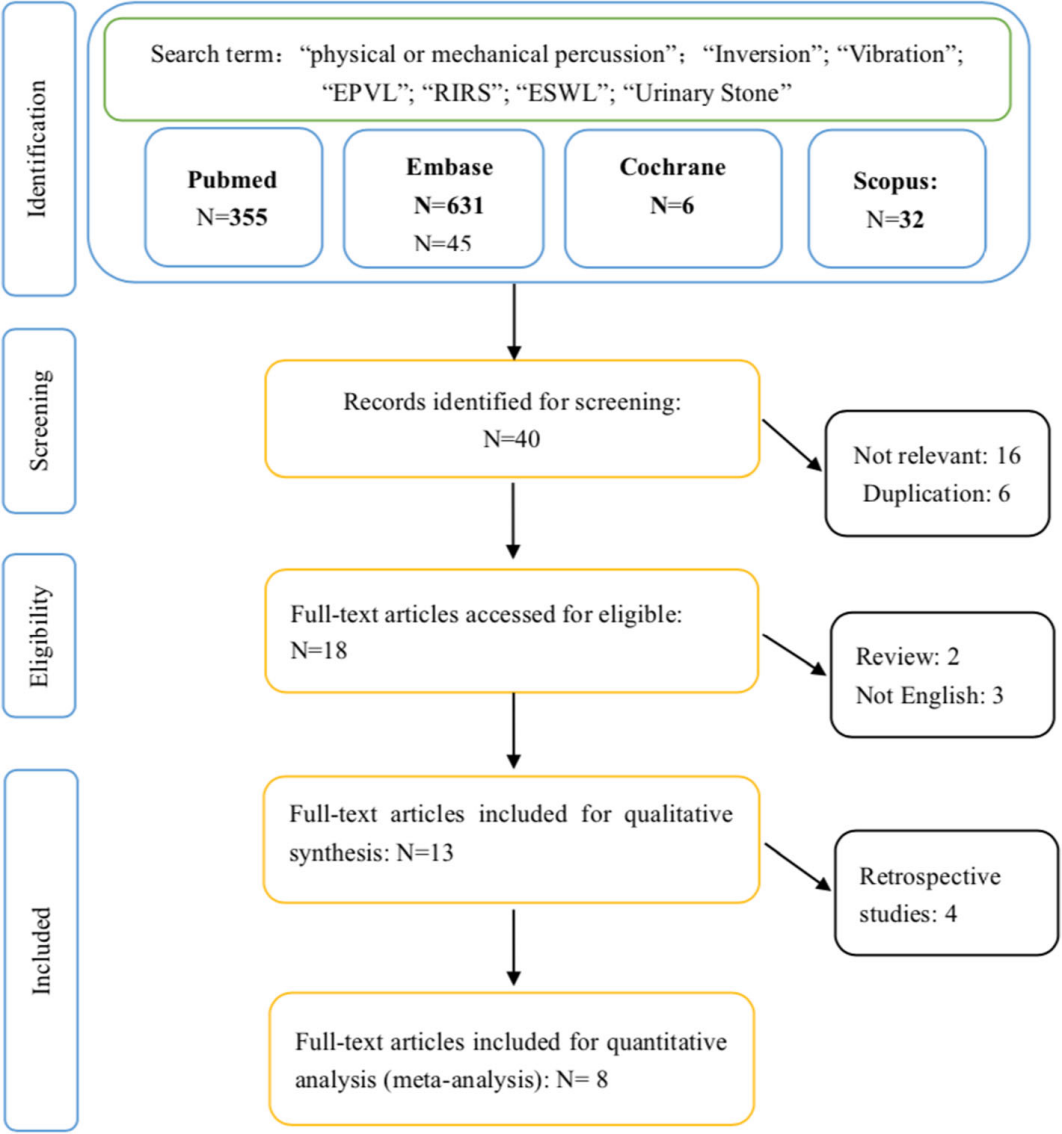
Flow of studies selection for systematic review and meta-analysis

The protocol of the present study has been registered in PROSPERO (CRD42019130228), and was also reviewed and approved by the Ethics Committee of the first affiliated hospital of Guangzhou Medical University. As pictured in the flow chart of this study in Fig. 1, systematic literature review in PubMed, Scopus, Cochrane library and Embase was performed in March 2019. A comprehensive literature search was conducted separately with following search strategy: (“physical or mechanical percussion”, OR “inversion”, OR “vibration”, OR “external physical vibration lithecbole”, OR “EPVL”), and (“extracorporeal shockwave lithotripsy”, OR “ESWL”, OR “flexible ureteroscopy”, OR “RIRS”), and (“residual stone”, OR “stone fragment”, OR “urinary stone”). The selection of relevant studies was in accordance with protocol items of PRISMA (Preferred Reporting Items for Systematic Reviews and Meta-Analyses) guidelines. The potential eligible studies from the cited references in the enrolled papers were also assessed. All the processes were completed by two reviewers LJP and JJW, disagreements were resolved by consensus after consulting WZ and GHZ.

### Selection criteria

Studies were enrolled in the present meta-analysis if met the following inclusion criteria: (1) Prospective studies either randomized controlled trials (RCTs) or non-RCTs; (2) The study subjects compared physical therapies (external physical vibration lithecbole, mechanical percussion, inversion, position change or other similar means) with conservative non-interventions; (3) Patients received ESWL or RIRS before physical therapy; (4) More than 30 adult patients in each study; (5) Patients’ demographics data, stone location and stone size information was presented; (6) Published in English. Studies as below would be excluded: (1) Retrospective studies; (2) Conference abstracts, reviews or editorials; (3) Repeated publications; (4) Studies Published in other language rather than English.

### Statistical analysis

Methodology was accomplished using Review Manager Version 5.3 software and R software, version R 3.6.1 (https://www.r-project.org/). The level of evidence (LE) was assessed using the GRADE system. To further assess the methodological quality of the studies, Newcastle-Ottawa Scale (NOS) was used for non-randomized controlled trials (N-RCTs), while Jadad scale for RCTs.

Since the categorical variables of SFR and complications rate were our primary study subject, statistical analysis was carried out using odds risk (OR) and 95% confidence intervals (CIs). Heterogeneity was assessed using the Higgins I^2^ statistic (minimal heterogeneity: 0–30%, moderate heterogeneity: 31–50%, significant heterogeneity: > 50%) [31]. Random effect model would be used for pooled analysis if significant heterogeneity (I^2^ > 50%) was noted, otherwise fixed model would be used. Furthermore, contour-enhanced funnel plots, sensitivity analysis and subgroup pooled analysis were performed to test the potential publish bias and heterogeneity.

In subgroup analysis, normal physical therapy of percussion, diuresis and inversion (PDI), and typical physical therapy of external physical vibration lithecbole (EPVL) were classified. To analyze SFR alteration following time change, the SFR at the time points of post-lithotripsy 1 week, 2 weeks and 1 month were analyzed. In another subgroup analysis, available residual stone fragments location was classified into upper calyx stone (UCS), middle calyx stone (MCS), lower calyx stone (LCS), upper ureteral stone and renal pelvic stone (UPS), or else stone location prior to lithotripsy was used instead.

## Results

### Characteristics and quality of the included studies

As listed in Table 1, a total of 8 prospective studies published from 2001 to 2019 were finally enrolled into the present analysis [29, 32–38], including 7 RCTs and 1 prospective case control study. Three studies were multi-center studies (NCT02645708, NCT02643134, one not registered). One study was after RIRS and the rest 7 studies were after ESWL. A total of 1065 subjects were included in the present study, 528 in the physical therapy group, and 537 in control group.

**Table 1:** summary of comparative studies included

Study+years	Period	Type	Location	Surgery	StoneSize	Gender	Age	BMI	LE	Quality
**Wu1** 2017 [32]	2016–2016	RCT	Kidney	RIRS	< 17	66/21:66/24	47.1 + 1.0:46.9 + 1.2	24.5 + 0.3:24.1 + 0.3	1b	5
**Wu2** 2017 [33]	2015–2016	RCT	upper urinary	ESWL	< 15	56/20:52/25	42.9 + 1.5:42.7 + 1.3	23.6 + 0.3:23.8 + 0.3	1b	5
**Tao** 2018 [34]	2017–2017	RCT	upper ureter	ESWL	1–20	83/44:96/48	49.6 + 6.1:50.4 + 5.7	23.6 + 2.9:23.1 + 3.3	1b	5
**Long** 2016 [35]	2014–2014	RCT	LCS	ESWL	6–20	20/14:22/15	44 + 9.5:45.8 + 9.9	25.2 + 3.4:25.6 + 2.9	1b	3
**Jing** 2018 [36]	2015–2016	RCT	Ureter, kidney	ESWL	NA	43/17:49/11	38.7 + 10.7:38.2 + 10.6	24.1 + 2.9:24 + 2.6	1b	4
**Albanis** 2009 [37]	28 months	Pro	LCS	ESWL	20	37/13:39/11	39(19–70):36 (16–69)	NA	2b	8#
**Chiong** 2005 [38]	Since 2001	RCT	LCS	ESWL	4–20	50/9:30/19	49 (21–71);45 (23–72)	25.8 + 2.7:25.2 + 4.27	1b	4
**Pace** 2001 [29]	1999–2000	RCT	LCS	ESWL	< 4	23/12:29/5	52 + 11.6:40.6 + 22.4	NA	1b	3

*NA* not available, *N* blank, # nos score, *LCS* lower calyceal stone, *Pro* prospective study. Stone size (mm)

**Fig. 2:**
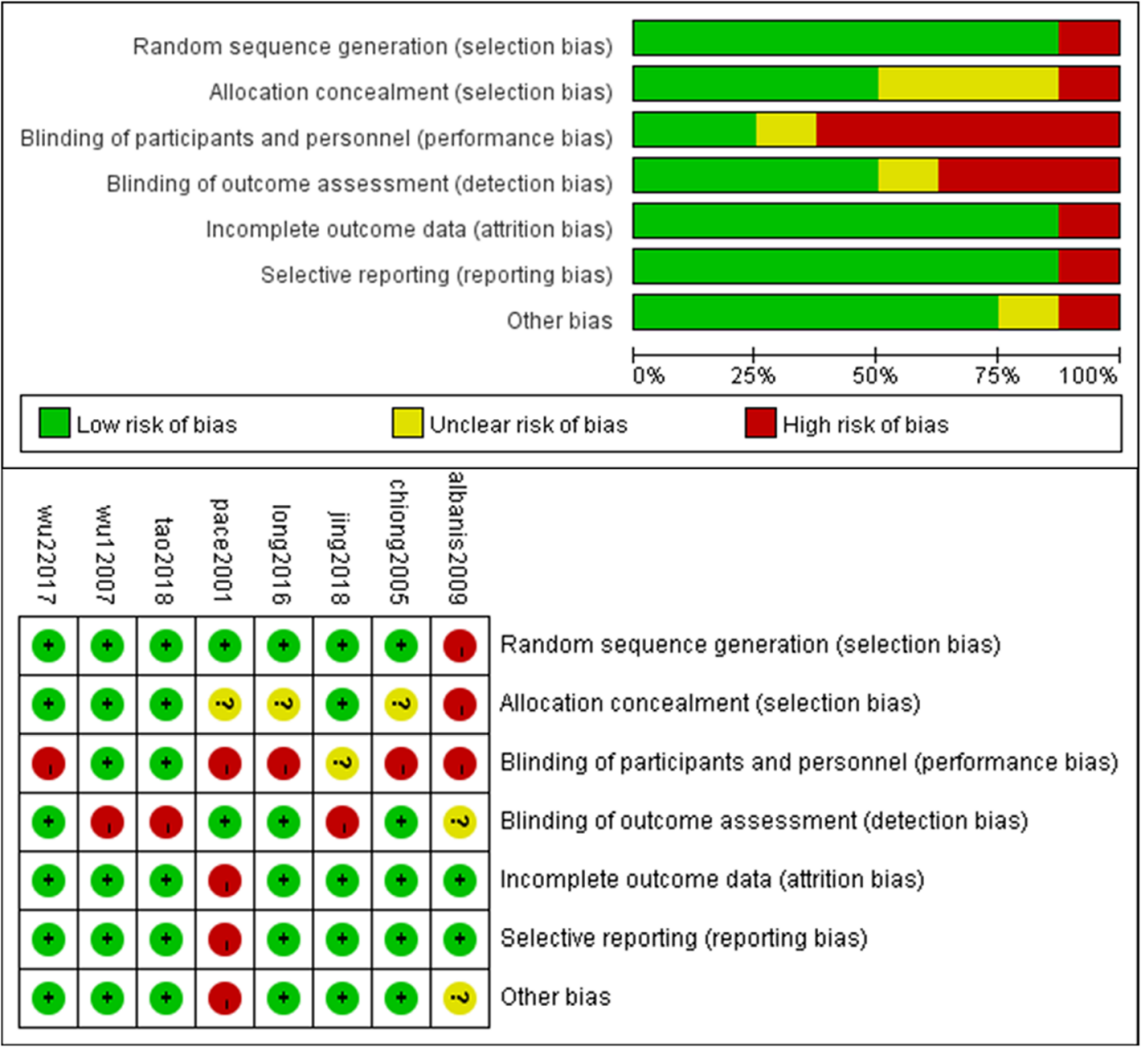
Risk of bias summary

The baseline information of gender (OR:0.96, 95% CI: 0.71–1.28, *p* = 0.76), age (MD: 0.17, 95% CI: − 0.11-0.46, *p* = 0.24) and BMI (MD: 0.17, 95% CI: 0.26–0.60, *p* = 0.45) were comparable in over population, details were showed in Table 1. The stone sizes were all less than 2 cm prior to lithotripsy, except not available in one study. The details of stone locations and stone fragments numbers after lithotripsy were not available in all the studies. And LE of all RCTs was 1b and the score of methodological quality ranged from 3 to 5. Risk of bias assessment was described in Fig. 2.

Details of different physical techniques, including the techniques name, machine used, time to first session physical therapy, time for each session of percussion, inversion angle, water drinking, whether medicine applied and the follow-up were presented in Table 2. To be specific, 4 studies with use of EPVL (external physical vibration lithecbole), 2 with PDI (percussion, diuresis and inversion), 1 with MP (mechanical percussion), and 1 with HDI (hydration diuresis and inversion). Discrepancies were noted in different PDI techniques, while EPVL had a similar protocol. Drinking 500-3000 ml water was advised in 7 studies prior to the physical therapy to promote stone expulsion by diuresis. As for the time to perform the first session physical therapy, 4 studies performed physical therapy immediately after ESWL, 3 within postoperative 1 week, and 1 in 3 months after ESWL. One to four sessions of physical therapy in total were performed. Combined with percussion, an inversion angle of 12–60° tilt was selected. Percussion parameter was definite in EPVL, with a power of 40 W, vibration frequency of 2800–3500 blows per minute, amplitude of 5 mm, while these parameters were not available or consistent in other physical therapy procedures. The percussion lasted 10–20 min for each session. The definition of stone free status was no non-intervention of stone fragments under radiography (KUB, CT or ultrasonography).

**Table 2:** Information of different physical treatments

Study	Technique	Machine	First treat	Percussion Time (min)	Inversion (degree)	Drinking (ml)	medicine	SF	Treat session	Follow Up
Wu1	EPVL	Friend I	1 W	16 ~ 20	26	1000–2000	No	0	1–2	2,3,5 W
Wu2	EPVL	Friend I	1 W	16–20	26	1500–2000	No	0	1–2	1,2,4 W
Tao	EPVL	Friend I	30 min	15–20	26	1000	NA	0	1	1,2,4 W
Long	EPVL	Friend I	IM	6_12	26	1000–1500	NA	0	1–4	1,3 W
Jing	MP	VT300	IM	15–20	35	1000–3000	NA	0	2/2d	1,2 W
Albanis	HDI	MPL 9000	IM	NA	12	1000	F 40 mg	0	4	1,3 M
Chiong	PDI	NA	1 W	10	45	500	NA	0	4/1-2w	3 M
Pace	PDI	NA	> 3 M	10	60	NA	F 20 mg	0	4/4w	3 M

*NA* not available, *N* blank, *IM* immediate, *W* week, *M* month, *F* Furosemide, *SF* definition of stone-free (mm)

### Meta-analysis results

#### Stone free rate

**Fig. 3:**
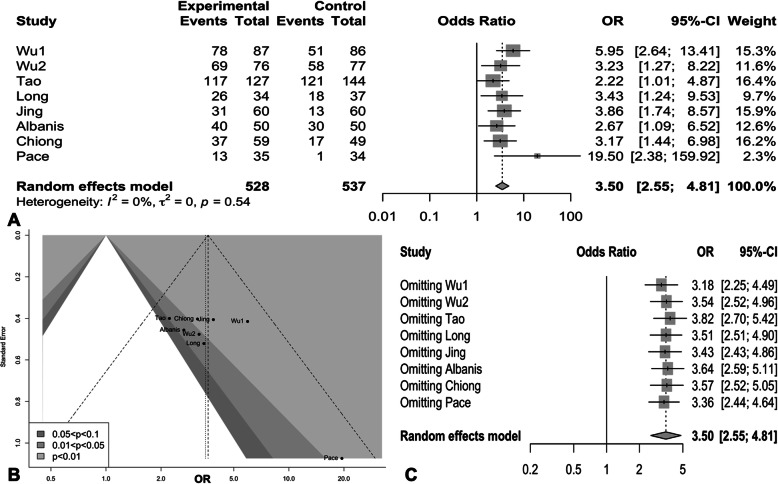
**a** Meta-analysis of stone-free rate among overall included studies. **b** Contour-enhanced funnel plots. **c** Results of sensitivity analysis

**Fig. 4:**
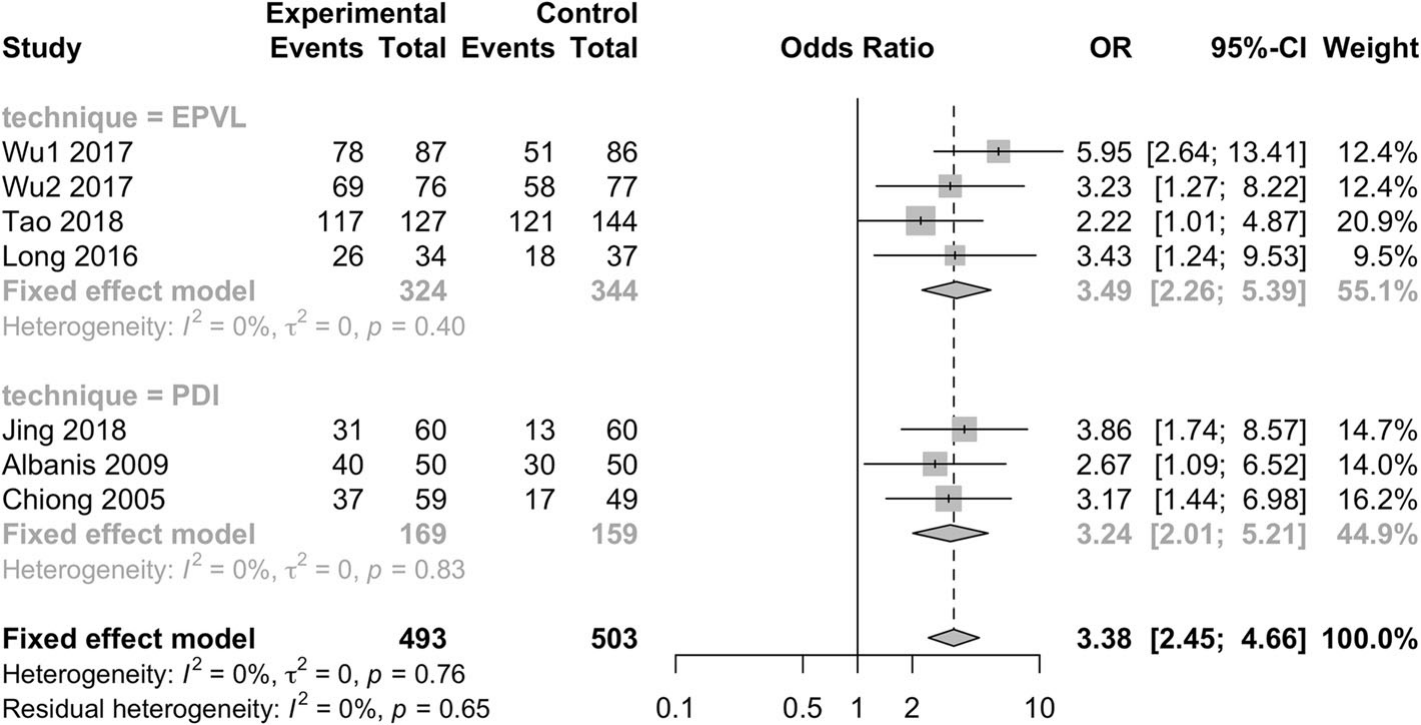
Meta-analysis of stone-free rate after excluding studies in bias and subgroup analysis of EPVL and PDI

**Fig. 5:**
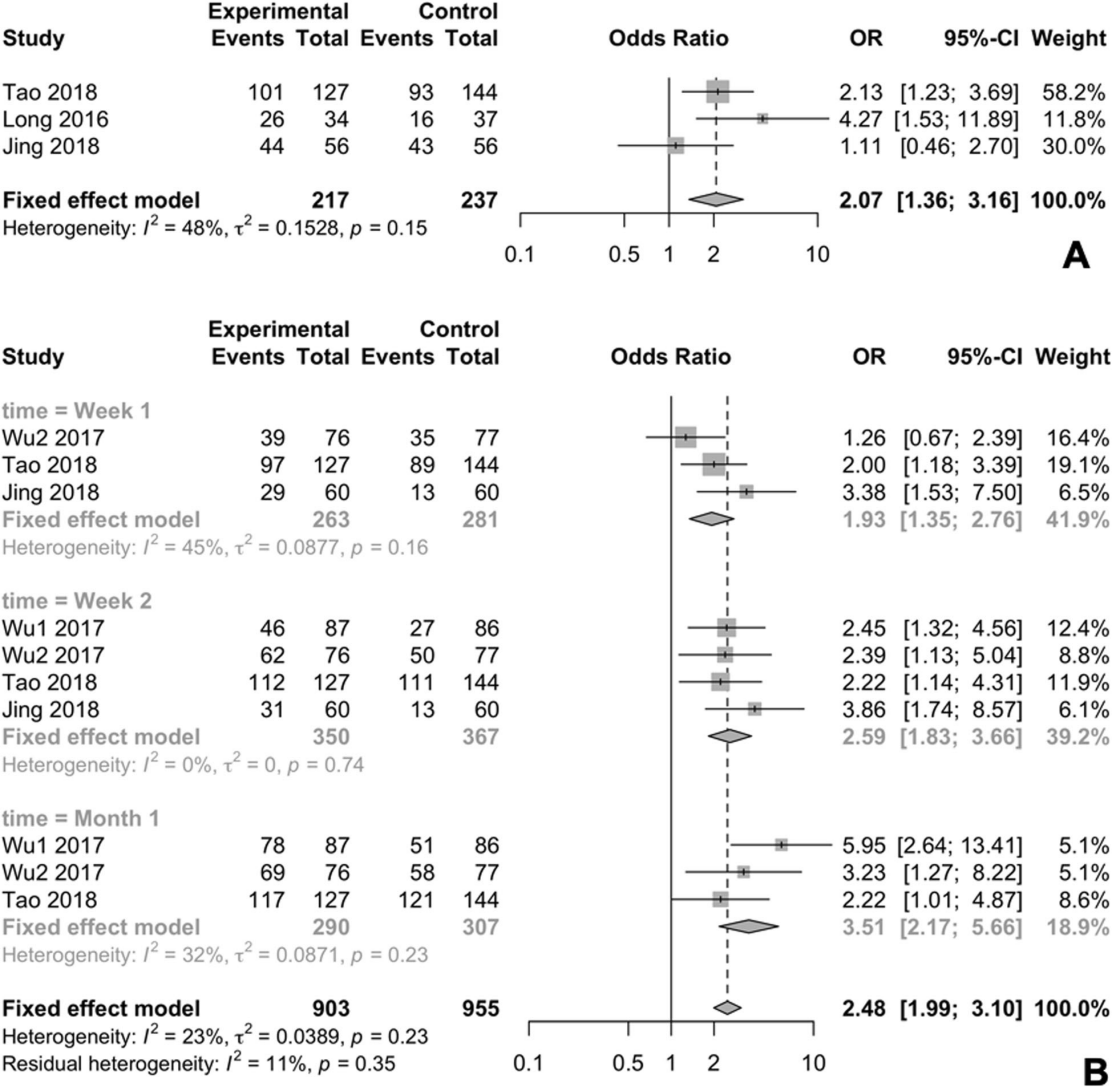
**a** Meta-analysis of first two-day stone expulsion. **b** Subgroup analysis of stone-free rate in first 2 weeks and first month

As shown in Fig. 3.A, a higher SFR was noted in physical therapy group (OR:3.50, 95% CI:2.55–4.81, *p* < 0.0001) than non-intervention group. There was no significant difference in heterogeneity (I^2^ = 0%, *p* = 0.54). Sensitivity analysis in Fig. 3.C showed that omitting any study would not change the final results or cause large elevation of quantitative difference. But minimal publication bias was detected in contour-enhanced funnel plots (t = 1.85, *p* = 0.113, Fig. 3.B), since the study of Pace et.al showed a marked deviation when compared with other studies. Given that the study of Pace et.al was performed in 2000, which was much earlier than others, and the results was far abnormal when compared to others, we decided to remove it from the final pool analysis. After excluding this study from final analysis, SFR in physical therapy group was still higher when compared to non-intervention group (OR: 3.38, 95%CI: 2.45–4.66, *p* < 0.0001), and there was no significant difference in heterogeneity (I^2^ = 0%, *p* = 0.76, Fig. 4). Additionally, higher rate of first two-day stone expulsion was observed in physical therapy group than non-intervention group (OR: 2.07, 95% CI: 1.36–3.16, *p* = 0.0007), as showed in Fig. 5a.

#### Complication

**Fig. 6:**
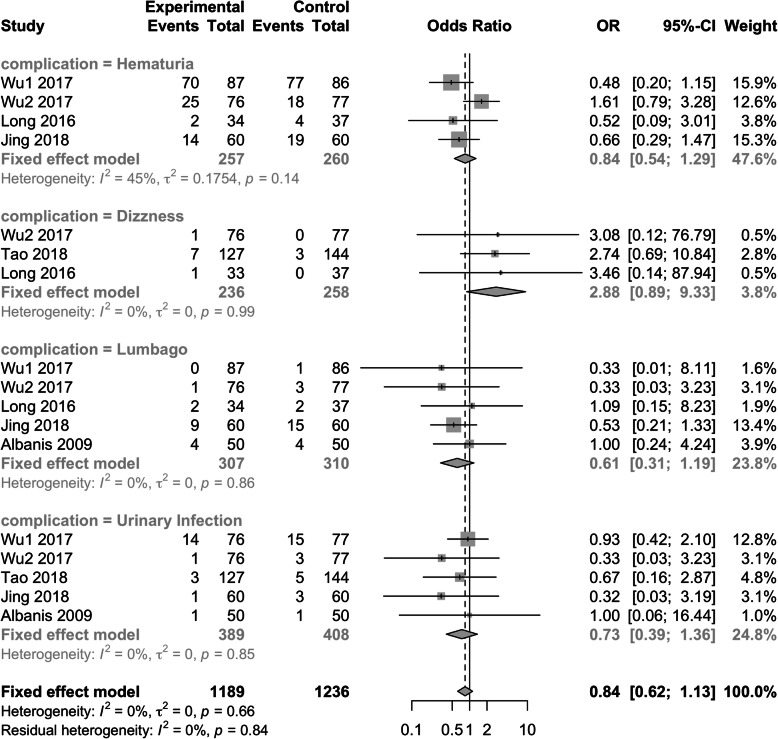
Meta-analysis of related complications

Overall complication rate was comparable as showed in Fig. 6 (OR: 0.84; 95% CI: 0.62–1.13; *p* = 0.237), there were no significant difference in terms of hematuria (OR: 0.84; 95% CI: 0.54–1.29; *p* = 0.423), dizziness (OR: 2.88; 95% CI: 0.89–9.39; *P* = 0.078), lumbago (OR: 0.61; 95% CI: 0.31–1.19; *P* = 0.146) and urinary tract infection (OR: 0.73; 95% CI: 0.39–1.36; *P* = 0.328), respectively.

### Subgroup meta-analysis

#### SFR in different physical therapy

As presented in Fig. 4, when different techniques of physical therapy were classified into EPVL and PDI, the SFR no matter in EPVL (OR: 3.47; 95% CI: 2.24–5.37; *p* < 0.0001) or in PDI (OR: 3.24; 95% CI: 2.01–5.21; *p* < 0.0001) was higher than SFR in non-intervention group.

#### SFR in different time point

As depicted in Fig. 5b, an overall higher SFR was observed in physical therapy group (OR: 2.48; 95% CI: 1.99–3.10; *p* < 0.0001) when compared to non-intervention group. Specifically, in the first week (OR: 1.93; 95% CI: 1.35–2.76; *p* = 0.0003), in second week (OR: 2.59; 95% CI: 1.83–3.66; *p* < 0.0001), and in first month (OR: 3.51; 95% CI: 2.17–3.10; *p* < 0.0001).

#### Influence of stone location to SFR

**Fig. 7:**
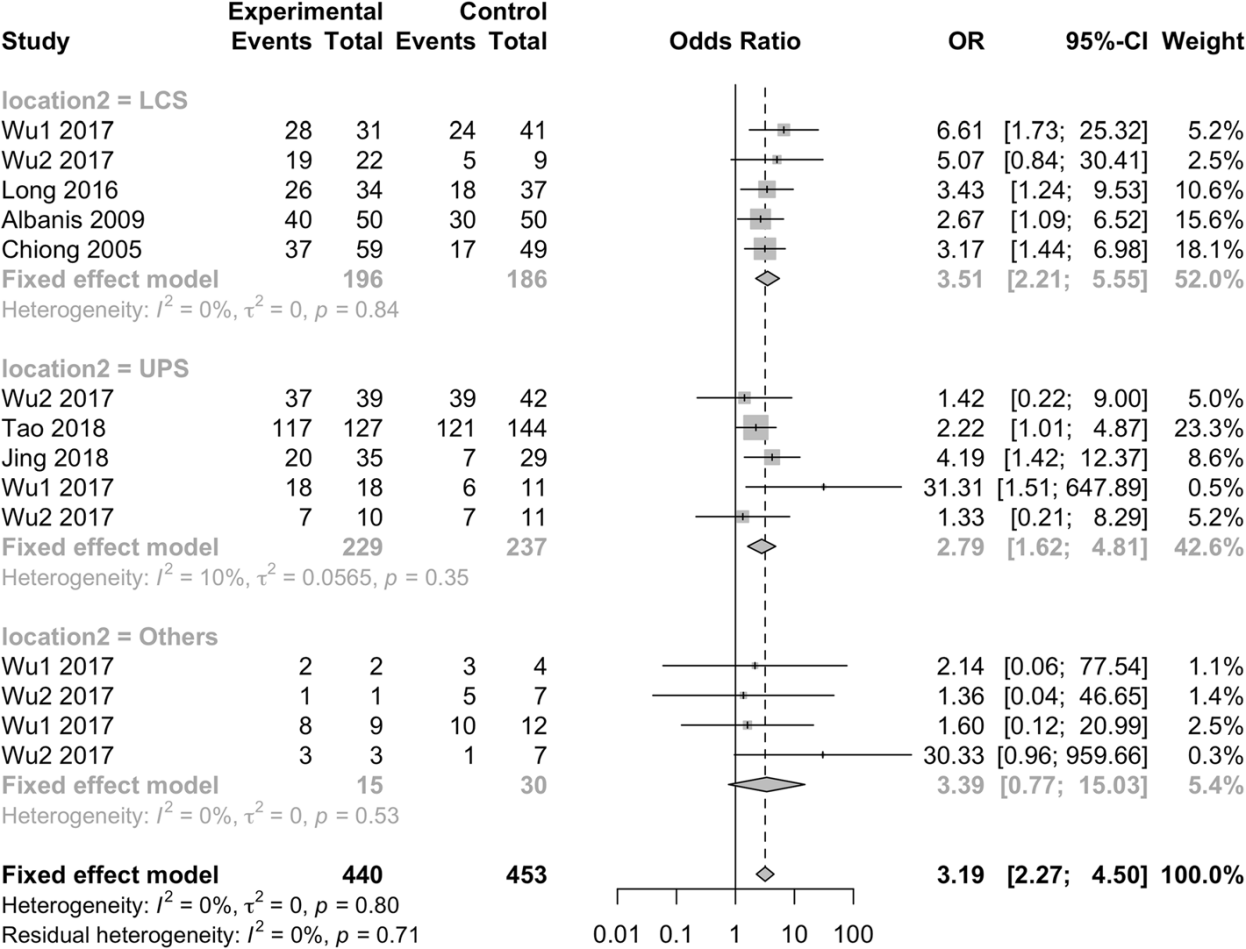
Subgroup analysis of influence of different stone fragment locations. LCS = lower calyx stone, UPS = upper ureter and renal pelvic stone, Others = upper calyx and middle calyx stone

A higher SFR in physical therapy group was noted in the overall analysis (OR: 3.19; 95% CI: 2.27–4.50; *p* < 0.0001) in terms of different stone locations. In the subgroup meta-analysis of the stone fragments location (Fig. 7), physical intervention group owned a higher SFR in LCS (OR: 3.51; 95% CI: 2.21–5.55; *p* < 0.0001) and UPS (OR: 2.79; 95% CI: 1.62–4.81; *p* = 0.0002). UCS and MCS were mixed into pooled analysis as others due to few information, no significant difference was noted (OR: 3.39; 95% CI: 0.77–15.03; *p* = 0.108).

## Discussion

Based on the present meta-analysis, physical therapy following RIRS and ESWL did improve the SFR at different time points (week1, week2 and month1). The stones in lower calyx (OR: 3.51; 95% CI: 2.21–5.55; *p* < 0.0001), upper ureter and renal pelvic (OR: 2.79; 95% CI: 1.62–4.81; *p* = 0.0002) benefited much more from the physical therapy than the stone in other locations (upper calyx and middle calyx). Furthermore, the physical therapy did not bring more complications (all *p* > 0.05).

As was known, a serial of factors would influence the spontaneous passage of the stone fragments following RIRS and ESWL, for example, lower pole stones, large stone burden and stone density parameters have been reported to affect the SFR significantly [39, 40]. Furthermore, others factors such as ureter condition, washing of urine, and ureteral smooth muscle movement should also be taken into consideration [41]. After ESWL and RIRS, what we can do to improve the SFR is that, facilitating the stone fragments move into ureter, pushing the fragments passage from the dilated ureter. Thus, self-help position therapy, diuresis and relaxation ureteral smooth muscle have been clinically tried [17, 25].

In 2000, Honey et.al [42] reported PDI (percussion diuresis and inversion) can effectively mobilize residual stones out of the lower calyx, and eventually passage. In a meta-analysis published in 2013 from Liu et.al, PDI was safe and effective to assist clearance of LCS after ESWL (OR: 0.62, 95% CI: 0.47–0.82). Owing to the limited number of studies enrolled in that meta-analysis, a pooled analysis was required to investigate the clinical outlook of physical therapy. Fortunately, the present meta-analysis enrolled enough studies and testified the effectiveness of PDI in improving SFR following ESWL and RIRS.

With the increasing experience and regeneration of equipment, a new device called EPVL was invented in China, which can provide a well-controlled inversion and body position changing from a rotating couch, and also a circled mechanical percussion [35]. With multiple approaches for mechanical percussion and effective percussion could be performed, EPVL was reported to improve the SFR following ESWL and RIRS. In 2016, Zhang et.al [43] performed a meta-analysis enrolling 5 randomized or Quasi-randomized controlled trials and demonstrated that, EPVL was effective in promoting upper urinary tract residual stones expulsion (OR:4.50, 95% CI:2.02–10.00, *p* = 0.0002). According to subgroup analysis of different techniques in the present meta-analysis, EPVL provided a higher SFR after ESWL and RRIS (OR: 3.47; 95% CI: 2.24–5.37; *p* < 0.0001), as well as PDI (OR: 3.24; 95% CI: 2.01–5.21; *p* < 0.0001). Given that there was no standard and widely acceptable protocol for physical therapy, EPVL might provide a relative uniformed and repeatable protocol for clinical practice, thus more practical than other physical therapies.

Zhang et.al [44] investigated the potential ideal time to perform EPVL after RIRS, in which 3 days, 7 days, 14 days after RIRS were compared. They found that, the best time to perform EPVL was 3 days after RIRS at all the time points (7 days, 14 days, 28 days), with a highest SFR of 91.11% in 28 days. Similarly, physical therapy provided a higher SFR (OR:3.38, 95%CI: 2.45–4.66, *p* < 0.0001) at all time points (week 1, week 2 and month 1) was noted in the present meta-analysis, but the ideal time point to performed the first session physical therapy was still not conclusive.

Medical expulsive therapy (MET), including diuretics, Chinese patent medicine, α-receptor blockers (tamsulosin) have been used as auxiliary procedure to improve SFR following lithotripsy procedures. But the role of tamsulosin in ureter dilation is still controversial, as well as other medicines. In a three-arm study, Liu et.al [30] compared EPVL combined tamsulosin, EPVL alone and tamsulosin alone, they found that, EPVL combined tamsulosin could get a higher SFR for distal ureter stones when compared to EPVL alone and tamsulosin alone in the first week (91.1% vs. 50.5% vs.50%, *p* < 0.05). However, there was no significant difference in final SFR (94.5, 93.5 and 93.6%, *p* > 0.05). Diuresis was supposed to help stone fragments expulsion through urine washing, studies enrolled in the present meta-analysis recommended enough water drinking (1000-3000 ml) before physical therapy. However, the volume of drinking water, when to drink, and the role of furosemide was still inconclusive, since limited information can get from the enrolled studies. Thus, further investigations were required to testify the role of combined MET in physical therapy.

When it came to the complications, we did not found any significant difference in terms of hematuria, lumbago and urinary infection between physical therapy and non-intervention (all *p* > 0.05). Even though EPVL and PDI did facilitate the stone fragments passage, but did not increase the risk of renal colic and steinstrasse formation in the present meta-analysis.

To be noticed, there were several limitations about this meta-analysis. The primary limitation was the small number of eligible studies and sample sizes. In addition, the ideal time point to performe the first session physical therapy, and the role of tamsulosin and diuresis in physical therapy was still inconclusive, which need further investigation.

## Conclusions

Physical therapy is effective in improving the SFR after ESWL and RIRS, especially for lower calyx stones, upper ureter and renal pelvic stones, while without significant side effects. External physical vibration lithecbole (EPVL) might provide a relative uniformed and repeatable protocol for clinical practice of physical therapy. Well designed and large sample RCTs are still needed to assess the details of physical therapy.

## Data Availability

All data generated or analyzed during this study are included in this published article and its supplementary information files.

## Abbreviations

SFR: Stone free rate
RIRS: Retrograde intrarenal surgery
ESWL: Extracorporeal shockwave lithotripsy
EPVL: External physical vibration lithecbole
PDI: Percussion, diuresis and inversion
LCS: Lower caliceal stones
UCS: Upper calical stones
MCS: Middle caliceal stones
UPS: Upper ureter and renal pelvic stone
MET: Medical expulsive therapy
